# Transcranial direct current stimulation: five important issues we aren't discussing (but probably should be)

**DOI:** 10.3389/fnsys.2014.00002

**Published:** 2014-01-24

**Authors:** Jared C. Horvath, Olivia Carter, Jason D. Forte

**Affiliations:** Psychological Sciences, University of MelbourneMelbourne, VIC, Australia

**Keywords:** transcranial direct current stimulation (tDCS), variability, reliability, efficacy, mechanisms of action

## Abstract

Transcranial Direct Current Stimulation (tDCS) is a neuromodulatory device often publicized for its ability to enhance cognitive and behavioral performance. These enhancement claims, however, are predicated upon electrophysiological evidence and descriptions which are far from conclusive. In fact, a review of the literature reveals a number of important experimental and technical issues inherent with this device that are simply not being discussed in any meaningful manner. In this paper, we will consider five of these topics. The first, *inter-subject variability*, explores the extensive between- and within-group differences found within the tDCS literature and highlights the need to properly examine stimulatory response at the individual level. The second, *intra-subject reliability*, reviews the lack of data concerning tDCS response reliability over time and emphasizes the importance of this knowledge for appropriate stimulatory application. The third, *sham stimulation and blinding*, draws attention to the importance (yet relative lack) of proper control and blinding practices in the tDCS literature. The fourth, *motor and cognitive interference*, highlights the often overlooked body of research that suggests typical behaviors and cognitions undertaken during or following tDCS can impair or abolish the effects of stimulation. Finally, the fifth, *electric current influences*, underscores several largely ignored variables (such as hair thickness and electrode attachments methods) influential to tDCS electric current density and flow. Through this paper, we hope to increase awareness and start an ongoing dialog of these important issues which speak to the efficacy, reliability, and mechanistic foundations of tDCS.

## Introduction

Transcranial direct current stimulation (tDCS) is currently being promoted as a cheap and effective tool to enhance cognitive and behavioral function. A recent surge of public interest in this device (evidenced by several tDCS devices appearing on the public market) has doubtless been driven by the belief that these enhancement claims are robust, reliable, and well elucidated. However, the research exploring the efficacy of tDCS is far from conclusive.

It's commonly assumed that tDCS shifts the resting membrane potential and synaptic strength of neurons in a predictable and consistent manner. More specifically, hypo-polarization of neurons under the anodal electrode is believed to increase the likelihood of their firing, whilst hyper-polarization of neurons under the cathodal electrode is believed to decrease the likelihood of their firing [for an in depth mechanistic overview, see, Stagg and Nitsche (2011)]. Similar to efficacy, however, a close inspection of the literature reveals short-comings of the anode excite/cathode inhibit model.

In response to efficacy and mechanistic uncertainties, many practitioners focus on the manipulation of three adjustable tDCS parameters: current density, electrode position, and stimulation duration. Whereas these three variables certainly play a large role in tDCS outcomes, there are a number of equally important issues relevant to both efficacy and mechanism that simply are not being discussed in any meaningful manner.

In this paper, we will explore five notable indicators and/or sources of inconsistency associated with the use of tDCS in the current literature: inter-subject variability, intra-subject reliability, lack of effective sham and blinding protocols, motor and cognitive interference, and electric current influences. Throughout this piece, we will draw examples solely from studies which explore the effects of tDCS over the motor cortex on MEP amplitude in healthy populations. We have chosen to do this for two reasons: first, MEP amplitude modulation is easily the most explored and reliably demonstrated outcome measure in the tDCS in the literature. Second, as the majority of neurophysiologic, clinical, and behavioral claims cite this work as mechanistically foundational, any issues apparent in this literature will necessarily be applicable to and concern any other outcome measure utilized.

## Inter-subject variability

tDCS must demonstrate similar (or comparable) effects across a range of people before it can be meaningfully applied in healthy and/or clinical populations. However, a survey of the literature reveals extensive between- and within-group variation suggestive of an inconsistent effect between individuals.

As an example of large between-group variation, Fricke et al. (2011) recently reported data from two different groups that underwent an identical stimulation protocol (0.0286 mA/cm^2^ current density; anode M1/cathode contralateral orbit montage; 5 min duration). Whereas one group demonstrated an average MEP amplitude enhancement of 93.2% in the 5 min following tDCS, the second group demonstrated an average MEP amplitude enhancement of only 9.2%: a between-group difference of 913%. Similarly, in two different studies from 2004 using identical stimulation protocols (0.0286 mA/cm^2^ current density; cathode M1/anode contralateral orbit montage; 9 min duration), Nitsche et al. reported 30 min group MEP amplitude inhibitions of 42.9% (Nitsche et al., 2004a) and 20.0% (Nitsche et al., 2004b): a difference of 110%. Even more variable, these researchers has reported group MEP amplitude enhancements following identical stimulation protocols (0.0286 mA/cm^2^ current density; anode M1/cathode contralateral orbit montage; 13 min duration) ranging from 54.4% (Nitsche et al., 2003a) to 19.3% (Nitsche et al., 2009); a difference of 184%.

Specific examples of within-group variability (beyond common deviation and/or error measures) are harder to come by as very few studies include individual data with their reports. However, of the few that have, the results are illuminating. For instance, following 9 min of anodal stimulation (0.0286 mA/cm^2^ current density; M1/contralateral orbit montage), Nitsche and Paulus (2001) reported one subject who demonstrated an incredible 295% increase in MEP amplitude and a second who demonstrated a weak 5% increase. More recently, following 20 min of anodal stimulation (0.06 mA/cm^2^ current density; M1/Contralateral orbit montage), Tremblay et al. (2013) reported one subject who demonstrated a 251% increase in MEP amplitude and a second who demonstrated a 41% *decrease* (see also, Roche et al., 2011).

One potential explanation for this extreme between- and within-group variability is the difficulty in properly and reliably targeting TMS pulses during lengthy protocols (Herwig et al., 2001; Sparing et al., 2008; Ahdab et al., 2010). Although modern MRI guided neuronavigation systems can be used to ensure accurate coil positioning across time, many tDCS studies have not utilized (or do not report utilizing) these systems. As such, it is possible subtle variation in coil placement and orientation with time may influence response variation.

A second potential explanation for this extreme between- and within-group variability is that tDCS generates differential response at the individual level which is masked by group averaging. An individual's unique neurophysiology, anatomy, and psychology may influence his/her response to tDCS. In fact, recent modeling work suggests parameters such as skull thickness, subcutaneous fat levels, cerebrospinal fluid density, and cortical surface topography can greatly influence current flow and density patterns during stimulation (Datta et al., 2012; Truong et al., 2013). As such, elucidation of individual and environmental influences on tDCS is necessary and may only be possible by looking at response characteristics (and related correlative factors) at the individual level.

## Intra-subject reliability

Beyond individual response patterns, it must be demonstrated that people respond in a similar and predictable manner to repeated sessions of tDCS before this tool can be meaningfully applied. Unfortunately, to our knowledge, response *reliability* at the level of the individual has not been explored (or, at least, reported) in the literature to date.

Of the (only) four studies which have explored group effects of tDCS on MEP amplitude in healthy populations across multiple days, two suggest response patterns may be reliable and replicable. Alonzo et al. (2012) explored the effects of anodal stimulation (0.0571 mA/cm^2^ current density; M1/contralateral orbit montage; 20 min duration) on MEP amplitude over the course of 5 days (Monday–Friday). Although these researchers reported variable baseline levels across the week, the ratio of pre- to post-stimulation group average MEP amplitudes did not significantly change from day-to-day. Using a similar protocol, Gálvez et al. (2013) reported similar findings: namely, whereas baseline levels changed throughout the week, the group averaged after-effects of daily stimulation did not significantly vary across 5 days.

Interestingly, the remaining two studies to explore group effects of tDCS on MEP amplitude in healthy populations across multiple days suggest response patterns may be unreliable and unpredictable. Monte-Silva et al. have twice looked at the effects of two sessions of tDCS on MEP amplitude with a 24 h block between sessions (Monte-Silva et al., 2010, 2012). In the first study (0.0286 mA/cm^2^ current density; cathodal M1/anodal contralateral orbit montage; 9 min duration), these researchers reported significantly reduced modulation of MEP amplitude following the second session of stimulation. More concerning, in the second study (0.0286 mA/cm^2^ current density; anodal M1/cathodal contralateral orbit montage; 13 min duration) these researchers reported not only a significant reduction in MEP amplitude modulation following the second session of stimulation, but also a reverse in modulation direction (inhibition rather than excitation following anodal stimulation) and unpredictable timing effects.

Considerably more data investigating effects across time is required before concluding tDCS is a reliable device. As individual response reliability is explored, however, it will be important to remember that intra-subject variability may not, in itself, suggest tDCS is unreliable. It is likely that circadian, metabolic, and hormonal cycles will differentially impact response. In fact, several researchers have already shown that stages of the menstrual cycle and cortisol levels impact plastic response to varied TMS protocols (Smith et al., 1999; Inghilleri et al., 2004; Sale et al., 2008, 2010). In addition, proper and reliable TMS coil positioning during lengthy protocols may also impact response variability (see above). With this in mind, it will certainly be informative to identify the factors that might influence unique tDCS response and whether these factor, themselves, modulate response in a reliable and predictable manner.

## Sham stimulation and blinding

If the dichometric anode excite/cathode inhibit mechanism of tDCS is valid, then comparing the polarities to each other makes determining the true effect of each extremely difficult (as one can never be certain the exact contribution of each polarity to the overall difference). Although practitioners aware of this comparative shortcoming extol the use of various control stimulation procedures (such as *sham* or *off-target active* stimulation), these procedures have not always proven effective or reliable across varied tDCS protocols (Ambrus et al., 2012; Brunoni et al., 2013; Davis et al., 2013; Palm et al., 2013). In addition, not nearly as many researchers have utilized control conditions as one might expect. In fact, of the 80 studies published to date exploring the effect of 0.0286 mA/cm^2^ tDCS current density with an M1/Orbit electrode placement on MEP modulation (the most utilized protocol in the literature), only 10 have compared results to a control condition (Table 1). This means 87.5% of the studies examining the foundational claim upon which the modern tDCS field is built have not utilized a proper control condition.

**Table 1:** **Studies exploring the effects of 0.0286 mA/cm^2^ current density, M1/Contralateral Orbit tDCS montage on TMS elicited MEP amplitude of intrinsic hand muscles at rest in healthy participants**.

Study	*N*	tDCS duration	Anode	Cathode	Control
Nitsche and Paulus, 2000 (*x4*)	10 and 9 (*x1*)/12 (*x2*)	4 s (*x1*)/5 min (*x3*)	**X** (*x4*)	**X** (*x4*)	**–**
Nitsche and Paulus, 2001 (*x5*)	12 (*x5*)	5, 7, 9, 11, and 13 min (*x1*)	**X** (*x5*)	**–**	**–**
Liebetanz et al., 2002	11	5 min	**X**	**X**	**–**
Nitsche et al., 2003a (*x3*)	12 (*x1*)/10 (*x2*)	4 s (*x1*)/9–13 min (*x2*)	**X** (*x3*)	**X** (*x3*)	**–**
Nitsche et al., 2003b (*x3*)	12 (*x3*)	5, 7, and 9 min (*x1*)	**–**	**X** (*x3*)	**–**
Lang et al., 2004a	8	10 min	**X**	**X**	**–**
Lang et al., 2004b (*x2*)	5 and 10 (*x1*)	10 min (*x2*)	**X** (*x2*)	**X** (*x2*)	**X** (*x1*)
Siebner et al., 2004 (*x2*)	5 and 8 (*x1*)	10 min (*x2*)	**X** (*x2*)	**X** (*x2*)	**X** (*x1*)
Nitsche et al., 2004a (*x4*)	6 (*x3*)/12 (*x1*)	4 s (*x1*)/7, 9–13 min (*x1*)	**X** (*x4*)	**X** (*x4*)	**–**
Nitsche et al., 2004b (*x3*)	12, 9, and 10 (*x1*)	4 s (*x1*)/5, 9–11 min (*x1*)	**X** (*x3*)	**X** (*x3*)	**–**
Nitsche et al., 2004c	12	9 min C/13 min A	**X**	**X**	**–**
Quartarone et al., 2004 (*x2*)	7 and 21 (*x1*)	5 min (*x2*)	**X** (*x1*)	**X** (*x1*)	**–**
Quartarone et al., 2005	8	10 min	**X**	**X**	**X**
Nitsche et al., 2006	12	9 min C/13 min A	**X**	**X**	**–**
Power et al., 2006	10	10 min	**X**	**X**	**X**
Nitsche et al., 2007a (*x8*)	12 (*x8*)	4 s (*x3*), 7 min (*x3*) 10 min (*x2*)	**X** (*x8*)	**X** (*x8*)	**–**
Nitsche et al., 2007b	12	7 min	**X**	**X**	**–**
Kuo et al., 2007	7	9 min C/13 min A	**X**	**X**	**–**
Antal et al., 2007	12	10 min	**X**	**X**	**–**
Boros et al., 2008	17	13 min	**X**	**–**	**–**
Kuo et al., 2008	7	9 min C/13 min A	**X**	**X**	**–**
Nitsche et al., 2009	12	9 min C/13 min A	**X**	**X**	**–**
Monte-Silva et al., 2009 (*x4*)	12 (*x4*)	9 min C/13 min A (*x4*)	**X** (*x4*)	**X** (*x4*)	**–**
Monte-Silva et al., 2010 (*x2*)	12 (*x2*)	9 and 18 min (*x1*)	**–**	**X** (*x2*)	**–**
Bradnam et al., 2011	18	15 min	**–**	**X**	**X**
Fricke et al., 2011 (*x4*)	8–12 (*x4*)	5 min (*x2*), 7 and 10 min (*x1*)	**X** (*x4*)	**X** (*x4*)	**–**
List et al., 2011	12	10 min	**–**	**X**	**–**
McCambridge et al., 2011	7 Active/5 Sham	10 min	**–**	**X**	**X**
Munneke et al., 2011 (*x3*)	10 (*x3*)	7, 11, and 15 min (*x1*)	**–**	**X** (*x3*)	**–**
Scelzo et al., 2011	12	13 min	**X**	**X**	**–**
Thirugnanasambandam et al., 2011	16	20 min	**X**	**X**	**–**
Di Lazzaro et al., 2012	30	20 min	**–**	**X**	**–**
Hasan et al., 2012	18	9 min	**–**	**X**	**–**
Schade et al., 2012 (*x2*)	8 (*x2*)	5 min (*x2*)	**X** (*x2*)	**X** (*x2*)	**–**
Suzuki et al., 2012	9	10 min	**X**	**X**	**X**
Monte-Silva et al., 2012 (*x2*)	15 (*x2*)	13 and 26 min (*x1*)	**X** (*x2*)	**–**	**–**
Hasan et al., 2013	20	9 min	**–**	**X**	**–**
Batsikadze et al., 2013 (*x2*)	9 and 8 (*x1*)	20 min (*x2*)	**–**	**X** (*x1*)	**X** (*x1*)
Schabrun et al., 2012 (*x3*)	21 A, 9 C, 13 s	20 min (*x3*)	**X** (*x1*)	**X** (*x1*)	**X** (*x1*)
Simis et al., 2013	11	20 min	**X**	**–**	**X**
TOTAL			62	67	10

A sham condition from Quartarone et al. (2004) was excluded due to reporting MEP modulation during motor imagery only (not at rest). One study from Monte-Silva et al., 2009 was excluded due to presenting replication data. A, Anode; C, Cathode; S, Sham.

Studies include the placebo condition/s in any drug or device interaction studies.

Comparing each polarity to its own baseline level (rather than the opposing polarity) does little to address the underlying issues inherent with sham-less protocols. It is commonly acknowledged that MEP amplitude is naturally an extremely variable measure (in fact, Valls-Sole recently pointed out, “The amplitude of MEP to single pulse TMS is not usually employed as a measure of functional relevance because of its large variability and dependence on many technical factors”; p. 9, *in press*). As such, it can be assumed there will *always* be some shift away from baseline levels, regardless of intervention (or lack thereof). Accordingly, the utilization of a control condition to differentiate between natural fluctuation and tDCS engendered effects is imperative.

As O'Connell et al. (2012) recently pointed out, tDCS blinding (especially when sham stimulation is being utilized) is of utmost importance yet incredibly difficult to achieve. In fact, these authors reported that, during stimulation using a high current density (0.0571 mA/cm^2^), neither the practitioner nor participant was effectively blinded. Beyond this, observable vasodilation (typically over the right orbit) makes practitioner blinding difficult at any current density (see, Palm et al., 2013). Finally, clear sensorial differences between active and sham stimulation (primarily reported as itching, tingling, and/or burning) make blinding participants who undergo multiple conditions difficult at any current density (Davis et al., 2013). As with any scientific study, ineffective blinding may lead to a number of undesirable confounds, including expectation effects, on-the-fly protocol adjustments, and reporting/assessment biases.

In order to elucidate the effects of varied tDCS paradigms, it is essential to continue to amend current and create novel, more effective control conditions. In addition, until such time as more reliable control protocols are developed, it may be beneficial to test for and report blinding procedures and efficacy (or lack-thereof).

## Motor and cognitive interference

Several lines of research suggest that any active motor and/or cognitive activity undertaken during or following tDCS can negatively interfere with or altogether abolish the effects of stimulation. The failure of many practitioners to take account of and further characterize this evidence is concerning.

Quartarone et al. (2004) were the first to report evidence of this interference effect. This group explored MEP amplitude modulation following 5 min of tDCS during motor imagery (0.0286 mA/cm^2^ current density; M1/contralateral orbit montage). Whereas imagery (undertaken following stimulation) appeared to prolong the effects of cathodal stimulation, it abolished the effects of anodal stimulation. Despite this important finding (that the act of *thinking* about motor movement could potentially eliminate tDCS efficacy), this paper went largely ignored and is rarely cited.

Several additional studies have confirmed this interference effect. For instance, Antal et al. (2007) reported that a cognitive task (a combined mathematics, language, geography, and history questionnaire) undertaken during stimulation abolished the effects of both anodal and cathodal stimulation on MEP amplitude modulation (0.0286 mA/cm^2^ current density; M1/contralateral orbit montage; 10 min duration). In addition, a simple motor task (pushing around a ball) undertaken during stimulation led to an equivalent decrease in MEP amplitude following both anodal and cathodal stimulation. This equivalent drop suggests the motor activity (perhaps due to fatigue of the target muscle) abolished the effect of stimulation as well. More recently, Miyaguchi et al. (2013) reported that anodal tDCS (0.0571 mA/cm^2^ current density; bilateral M1 montage; 10 min duration) delivered with a concurrent non-exhaustive active or passive motor task (self initiated or machine initiated finger abduction-adduction) led to an equivalent MEP amplitude reduction as did undertaking the active motor task alone (without stimulation). Again, this suggests the motor task abolished the effect of stimulation (see also, Thirugnanasambandam et al., 2011).

Secondary evidence for an interference effect can be seen in the often reported diminished tDCS MEP amplitude modulation in voluntarily contracted muscles (common in non-hand targets) compared to resting muscles. For instance, in the 40 min following cathodal stimulation (0.0286 mA/cm^2^ current density; M1/contralateral orbit montage; 15 min duration), Bradnam et al. (2010) reported an average compound MEP decrease of 15.3% in the right infraspinitus (shoulder) when the muscle was at rest, and an *increase* of 1.3% when the muscle was active. Similarly, in the 60 min following anodal stimulation (0.0571 mA/cm^2^ density; M1/contralateral orbit montage; 10 min duration), Jeffery et al. (2007) reported an average MEP amplitude increase of 34.8% in the right tibialis anterior (leg) when the muscle was at rest, and an increase of only 25% when the muscle was activated. Again, these results suggest that motor activity undertaken immediately following stimulation can significantly reduce or eliminate the modulatory effects of tDCS.

These findings suggest that relatively simple and difficult to control for thoughts and/or behaviors may eliminate tDCS efficacy. Clinically, tDCS is often used as an adjunct to physical rehabilitation following stroke (for review, Johansson, 2011). If the aforementioned studies are correct, combining tDCS with motor training may eliminate any desired tDCS effect. This holds true for healthy populations as well. Oftentimes, during long-duration, off-line stimulatory protocols, participants are instructed to simply relax during tDCS. This relaxation can take the form of reading, texting, surfing the internet, doing homework, etc. Is it possible these seemingly innocuous activities are enough to negate or otherwise interfere with the effects of tDCS as well? Additionally, in experiments utilizing MRI to explore the effects of tDCS, stimulation is often given outside the scanning room (although several tDCS devices are now MR compatible). During these protocols, participants must walk back to the scanning room and re-enter the scanner following stimulation: a series of non-trivial motor actions which may, again, interfere with or abolish any tDCS effects.

If it's possible the effects of tDCS are too weak to manifest during typical human behavior, this is important to determine before more effort and funding are expended utilizing inappropriate protocols. Until such a time as this issue is clearly resolved, it is important practitioners minimize motor and cognitive activity during and immediately following tDCS and during any proceeding procedure (including TMS and/or MRI).

## Electric current influences

There are a number of variables which may influence current density and flow to a great extent that have simply not been discussed in the literature to date. Although, as noted above, countless papers have discussed optimal electrode positioning, current density, and stimulation duration for specific outcomes (for discussion, Paulus, 2011), these discussions never seem to evolve past these three parameters.

One variable which may impact current density and flow (but which has yet to be discussed in the literature) is hair thickness. Simply put: hair is not a conductor—it is an insulator. Measurements suggest dry hair (<7% 25% H_2_0 content) has a resistivity of approximately 3 × 10^12^ Ω/cm whilst wet hair (25% H_2_0 content) has a resistivity of approximately 6 × 10^6^ Ω/cm (Feughelman, 1997). To put that into perspective, skin (the contact surface in many tDCS modeling studies) has a resistivity of approximately 2.15 × 10^−2^ Ω/cm (see, Miranda et al., 2006; Wagner et al., 2007: Ω = Ohm: Note—lower resistivity values equate to higher conductance).

**Figure 1:**
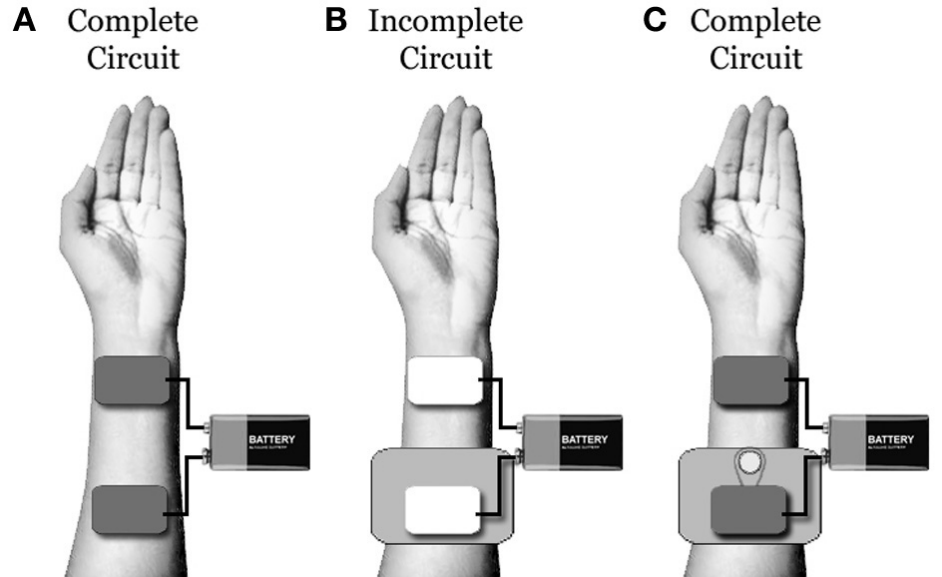
**(A) Under typical conditions, one will be able to build an electric circuit using two saline soaked tDCS sponges placed on a piece of clean skin. (B)** When a piece of non-conductive plastic is placed underneath one electrode, the circuit will be broken. **(C)** When a small hole is cut in the plastic and a small stream of saline is used to connect the skin under the hole to the electrode, the circuit will be re-built. This demonstrates that, even if a tDCS electrode is not in contact with the scalp, excessive saline can be used to bridge between the electrode and the skin. Unfortunately, with excessive saline, determining the location of the circuit connection and electrical density at this point is extremely difficult.

To combat this, practitioners often utilize large amounts of saline to saturate dense hair. Unfortunately, saline spread or dripping at the level of the scalp can guide current flow in undesirable and unpredictable directions. This fact can be easily demonstrated. First, place two saline soaked tDCS sponge electrodes on an easily accessible area of skin (such as the forearms or quadriceps). Next, place a piece of thick, non-conductive plastic under one of the electrodes to ensure no contact is made between the sponge and the skin. Under this set-up, you should be *un*able to complete the electric circuit. Now, cut a small hole in the plastic barrier (exposing the skin underneath), fill the small hole with saline, and run a continuous stream of saline between the hole and the sponge atop the plastic. Under this new set-up, you should be able to complete the electric circuit quite easily, regardless of how far away from the sponge you have made the small hole (if you are having trouble running a stream of saline between the electrode and the hole, you can substitute a thicker conductive gel: Figure 1).

This demonstration reveals that, even when there is no direct electrode/scalp contact (as may occur in participants with thick hair), excess saline can be used to bridge the tDCS current. However, when this is done, the precise location of the electric current entrance and/or exit points on the scalp will be largely unknown and unpredictable. In addition, when the electric current follows saline to the scalp, the current density also becomes largely unknown and unpredictable (as the number and size of contact points at the scalp becomes uncertain).

Sweat is a second often-ignored variable that may impact electric current dynamics. Because sweat increases skin conductivity (for review, Dawson et al., 2001), the amount of sweat on a participants scalp may influence current flow in important ways. It's possible that as salts, oils, and electrolytes accumulate in pores on the scalp, the skin will generate enough conductivity to ensure little or no current enters the cortex. However, aside from washing each subject's hair and ensuring a temperature controlled testing environment, how can this be accounted and/or controlled?

Finally, the means by which electrodes are held in place at the scalp may also influence electric current dynamics. For instance, several contemporary tDCS sponge electrodes include plastic rings at the corners (presumably anchor the electrode in place). Unfortunately, unless specifically manufactured, most plastics are non-conductive. Whether or not the plastic used in these electrodes has been produced to conduct electricity is uncertain, although vasodilation patterns following stimulation with these electrodes (which typically reveals no dilatory response underneath the plastic rings themselves) suggests they are not. This may impact current density and flow in unpredictable and uncontrollable ways. In addition, many practitioners hold sponge electrodes in place using rubber straps which are narrower than the electrodes themselves. With these straps, centralized pressure can cause the periphery of the electrodes to “flare” upwards reducing contact area (and, by extension, increasing current density). Given the apparent variability seen within and between individuals (outlined above), it is important to properly consider the influence these (and other) factors may be having on response characteristics.

## Conclusion

Recently, several practitioners have noted concerns about modern tDCS conceptions and mechanistic models (Bikson, 2013; Paulus et al., 2013). In addition, a number of studies have also begun to explore response variation in response to adjustments in current density, electrode position, and/or stimulation duration (Im et al., 2008; Bikson et al., 2010; Bastani and Jaberzadeh, 2013a,b). Although doubtless important, this work does not address the larger foundational issues raised in this paper.

Although we have chosen to focus on tDCS, many of the issues examined in this paper are applicable to other non-invasive modulatory tools; such as transcranial alternating current stimulation (tACS) and transcranial random noise stimulation (tRNS). These devices are often modified tDCS devices and the protocols utilized by each are often modeled after modern tDCS protocols. Because of this, although there is not enough data in the literature to confidently discuss response variability and reliability, issues of blinding, interference, and electric current influences are highly relevant to these novel tools.

If the field of tDCS is to avoid becoming a footnote in the annals of neuroscientific research, it is time to collectively acknowledge there are shortcomings in our current understanding of this device, its functional parameters, its general efficacy, and its reliability. Rather than seeing the aforementioned issues as a detriment to the field, we should use them to guide future research and exploration. For instance, acknowledging variability can encourage us to explore individual response patterns (and correlate these with related secondary measures to tease-out possible state-dependency effects). Acknowledging the lack of effective sham and blinding techniques can encourage us to develop better more effective devices. Acknowledging the interference effect of motor and/or cognitive activity can inspire us to devise more comprehensive protocols. It is hoped that increased awareness and open discussion of these important issues will lead to a more rigorous and accurate foundation upon which tDCS can be developed into the future.

### Conflict of interest statement

The authors declare that the research was conducted in the absence of any commercial or financial relationships that could be construed as a potential conflict of interest.
